# Sleep Outcomes With Cognitive Behavioral Therapy for Insomnia Are Similar Between Older Adults With Low vs. High Self-Reported Physical Activity

**DOI:** 10.3389/fnagi.2018.00274

**Published:** 2018-09-13

**Authors:** Timothy Yeung, Jennifer L. Martin, Constance H. Fung, Lavinia Fiorentino, Joseph M. Dzierzewski, Juan C. Rodriguez Tapia, Yeonsu Song, Karen Josephson, Stella Jouldjian, Michael N. Mitchell, Cathy Alessi

**Affiliations:** ^1^Washington & Jefferson College, Washington, PA, United States; ^2^Geriatric Research, Education and Clinical Center, VA Greater Los Angeles Healthcare System, North Hills, CA, United States; ^3^Department of Medicine, David Geffen School of Medicine, University of California, Los Angeles, Los Angeles, CA, United States; ^4^Department of Psychiatry, University of California, San Diego, San Diego, CA, United States; ^5^Department of Psychology, Virginia Commonwealth University, Richmond, VA, United States; ^6^Department of Medicine, Pontificia Universidad Catolica de Chile, Santiago, Chile

**Keywords:** insomnia, chronic illness, veterans, older adults, cognitive behavioral therapy for insomnia, physical activity

## Abstract

We examined whether baseline self-reported physical activity is associated with the efficacy of cognitive behavioral therapy for insomnia (CBT-I) in older veterans. Community-dwelling veterans aged 60 years and older with insomnia received CBT-I in a randomized controlled trial. Participants who received active treatment were divided into low and high physical activity based on self-report. Sleep outcomes were measured by sleep diary, questionnaire and wrist actigraphy; collected at baseline, post-treatment, 6-month and 12-month follow-up. Mixed-effects models compared differences between physical activity groups in change in sleep outcome from baseline to each follow-up, and equivalence tests examined if physical activity groups were clinically equal. There were no significant differences in sleep outcomes between physical activity groups. Equivalence tests suggested possible equality in physical activity groups for five of seven sleep outcomes. Efficacy of CBT-I in older veterans was not associated with self-reported physical activity at baseline. Older adults with insomnia who report low levels of physical activity can benefit from CBT-I.

## Introduction

Aging is associated with both an increased prevalence of insomnia (Schubert et al., 2002; Ancoli-Israel and Martin, 2006) and a greater prevalence of decline in physical abilities and decreased participation in activities (Hung et al., 2011). Research suggests that at least one-third of older adults show some symptoms of insomnia (Schubert et al., 2002). Insomnia in older adults commonly occurs comorbidly with other mental and/or physical illnesses (Foley et al., 2004; Jaussent et al., 2011; Zhuang et al., 2016). In addition to being common, insomnia is an important condition that contributes to increased healthcare and other costs (Walsh and Engelhardt, 1999). In addition, decreased physical activity is common in older adults, with only approximately 40% of adults aged 75 years and older reporting that they regularly exercise (Centers for Disease Control and Prevention, 2017). This lack of physical activity has been estimated to be the cause of 3.2 million deaths each year (World Health Organization, 2013). Physical activity is considered beneficial for good health and has been used to treat a variety of conditions including depression, anxiety and other conditions (Carek et al., 2011; Dinas et al., 2011).

Prior studies suggest that physical activity has a positive effect on sleep. Physical activity is considered dynamically related to sleep in older adults (Dzierzewski et al., 2014) and beneficial in preventing the development of poor sleep quality (Vaz Fragoso et al., 2015). Individuals who perform some physical activity (i.e., walking) report fewer insomnia symptoms and better sleep than those who do not (Edinger et al., 1993; Endeshaw and Yoo, 2016). For example, moderate-intensity exercise was found to improve subjective sleep measures in older adults with sleep problems (King et al., 2008). Although one study reported no association between physical activity and sleep in frail nursing home residents, many of whom had significant cognitive impairment (Alessi et al., 1995), exercise programs have been found to improve sleep quality in older adults with sleep problems who live in general community settings (King et al., 1997). In addition, another study found that inactive older adults with poor sleep quality had greater improvements after moderate-intensity physical activity in comparison to already-active participants (Buman et al., 2011). In general, studies show that physical activity improves sleep measures, but timing and other factors may affect response (Maculano Esteves et al., 2014).

Cognitive behavioral therapy for insomnia (CBT-I) has been shown to be highly effective for treatment of chronic insomnia in a variety of populations, including among older adults (Okajima et al., 2011). In fact, CBT-I is strongly recommended as a first-line treatment for chronic insomnia in all adults (Qaseem et al., 2016) because of its favorable safety profile and better long-term outcomes than sedative-hypnotic medications. Unfortunately, most adults with chronic insomnia are not referred for this effective treatment (Grandner and Chakravorty, 2017). Despite increased access to CBT-I, primary care providers still do not typically refer their patients with insomnia for this evidence-based therapy, and efforts to address factors that limit referral for CBT-I are needed (Ulmer et al., 2017).

Traditional components of CBT-I include sleep restriction, stimulus control and cognitive therapy; other components (such as relaxation therapy) may also be used. Taken together, CBT-I commonly involves approaches such as: (1) decreasing excessive time in bed awake; (2) limiting naps, which can impair the sleep drive, and increasing daytime alertness via increased daytime activity and light exposure; (3) re-establishing the association between the bed and sleep; (4) decreasing disruptive stimuli in the sleep environment; (5) improving general sleep habits; and (6) rectifying incorrect beliefs about sleep (Edinger and Means, 2005; Morin et al., 2006). These and other components are broadly based on the 3-P model of insomnia which posits that insomnia results from predisposing, precipitating, and perpetuating factors for insomnia (Spielman et al., 1987).

Understanding which patients are likely to respond to CBT-I may improve referral rates among primary care providers. Efforts to identify predictors of treatment response to CBTI in older adults have focused on demographic characteristics, psychiatric and medical comorbidities, and sleep characteristics; and have generally not addressed baseline physical activity and function (Troxel et al., 2013). One obstacle that may limit referrals for CBT-I among older adults is a belief among providers that older adults with physical limitations may be less likely to respond to treatment. Indeed, it is possible that low baseline physical activity may limit the older patient’s ability to adhere with some aspects of behavior change included in CBT-I, such as increasing daytime activities to avoid daytime napping and decreasing excessive time spent in bed.

Studies suggest that physical exercise is beneficial for sleep; however, to our knowledge, prior studies have not addressed whether baseline self-reported physical activity levels are a factor in patients’ ability to participate in and benefit from CBT-I. In the context of secondary analyses of findings from a larger randomized controlled trial testing CBT-I in older adults, we sought to examine the relationship between baseline self-reported physical activity and sleep outcomes in older adults who received the CBT-I intervention. Specifically, we were interested in examining: (a) whether low baseline physical activity among participants who received CBT-I impacts the efficacy of the CBT-I intervention; and (b) whether older adults with high vs. low baseline physical activity display clinically equivalent CBT-I outcomes. We hypothesized that: (a) low baseline physical activity would indeed impact the efficacy of CBT-I; and (b) participants who had higher levels of baseline physical activity would have better sleep outcomes compared to those who had lower levels of baseline physical activity; in other words we expected sleep effects of CBT-I in older adults with high vs. low baseline physical activity levels would not be clinically equivalent.

## Materials and Methods

### Trial Design

This study involved secondary analysis of data from the treatment group of a randomized controlled trial comparing CBT-I (provided by non-clinician sleep coaches using a structured manualized program and supervised by phone by a behavioral sleep medicine specialist psychologist) with a general sleep education control. The active treatment involved typical components of CBT-I (i.e., stimulus control, sleep restriction, cognitive approaches); it was not an exercise intervention. Participants were randomized to receive CBT-I in either a small group or individual format, or to the control condition. The main study findings and study methods have been previously described (Alessi et al., 2016). Briefly, participants in the study were community-dwelling veterans aged 60 years and older who had chronic (i.e., 3 or more months’ duration) insomnia disorder based on the diagnostic criteria of the International Classification of Sleep Disorders, Second Edition (ICSD-2; Sateia, 2005). By design, the study had few exclusionary criteria, to ensure enrollment of a sample of older adults generalizable to a typical clinical population, including those across the range of physical health and comorbidity. Structured assessments were performed at baseline and at post-treatment (i.e., after completion of the treatment phase), and at 6-month follow-up and 12-month follow-up. All assessments were performed by trained research staff who were blinded to group assignment, and who used structured questionnaires. As previously reported in Alessi et al. (2016), compared to controls, participants who received CBT-I (i.e., the treatment group) showed greater improvement in sleep diary measures (i.e., sleep onset latency, wake after sleep onset, total wake time, sleep efficiency) and sleep questionnaires (i.e., Pittsburgh Sleep Quality Index (PSQI), Insomnia Severity Index (ISI)), compared to controls.

In this current secondary analysis, we focused on findings among participants who were randomized to receive CBT-I, excluding participants who were randomized to receive the control condition. Specifically, we categorized these treatment group participants based on their self-reported baseline physical activity. Physical activity was measured based on the participant’s response to a query regarding their frequency of physical activity in a typical week. CBT-I treatment results for individuals who stated that they participated in physical activity on most days (four or more) during a typical week were compared to treatment results of those who stated that they participated in physical activity three or fewer days per week (Pate et al., 1995). The study was approved by the VA Greater Los Angeles Healthcare System Institutional Review Board, and all participants provided written informed consent (ClinicalTrials.gov Identifier: NCT00781963).

### Participants

Participants were recruited among outpatient veterans who had visited an urban VA healthcare system in the prior 18 months and who lived within 30 miles of the facility. Potential participants were identified from the VA national data warehouse and were mailed a 25-item survey to identify those with evidence of insomnia disorder according to ICSD-2 criteria. Respondents who met ICSD-2 diagnostic criteria for insomnia based on the survey, who reported their sleep problems were present for 3 months or longer, and who were willing to be contacted for research purposes were assessed by trained research staff for study eligibility by phone. Participants were excluded if they were unable or unwilling to participate in person, if they reported a diagnosis of sleep apnea or they screened positive for sleep apnea (defined by an apnea-hypopnea index of 20 or greater) in ambulatory testing performed as part of the research assessment process, or if they had severe medical or psychiatric illness (Alessi et al., 2016). In the larger randomized controlled trial, 159 participants were randomized 1:1:1 to one of three groups (group treatment CBT-I, individual treatment CBT-I, or the control condition). There were no differences in outcomes between participants randomized to either group or individual CBT-I (Alessi et al., 2016), so these two active treatment groups were combined into one CBT-I treatment group for analyses. The current analysis used data collected from participants in the active treatment group (combining participants randomized to group or individual CBT-I; *N* = 106).

### Group Conditions

In this analysis, we were interested in self-reported physical activity, as might be reported to a primary care provider. The physical activity groups were determined based on response to the baseline questionnaire item, “During a typical week, how many days do you exercise or do physical activities such as take a walk, swim, garden, play a sport?” Participants who responded more than 3 days (majority of days) were placed into the high physical activity group, whereas subjects who responded 3 days or fewer were placed into the low physical activity group (Pate et al., 1995).

### Participant Characteristics

The characteristics of the participants including age, sex, race, level of education, marital status, employment status and amount of weekly physical activity were collected at baseline. Additional measures included items to determine self-reported comorbidities, which were totaled and reported as a comorbidity index (Selim et al., 2004). Pain was measured using the seven-item Geriatric Pain Measure (GPM) pain intensity subscale (Ferrell et al., 2000). Depression was assessed using the Patient Health Questionnaire-9 (PHQ-9) total score (Kroenke et al., 2001). Health-related quality of life was measured using the Medical Outcomes Study 12-item Short-form Survey v2 (Ware et al., 2007), Mental and Physical Composite Subscales (SF12-MCS and SF12-PCS). Other measures included the Epworth Sleepiness Scale (ESS; Johns, 1991), the Flinders Fatigue Scale (FFS; Gradisar et al., 2007), and the Dysfunctional Beliefs and Attitudes about Sleep scale (DBAS-16; Morin et al., 2007).

### Outcome Measures

We focused on seven key sleep outcomes measured subjectively (using questionnaires and sleep diaries) and objectively (using wrist actigraphy). Participants completed structured sleep questionnaires (which were collected by trained research staff in participant interviews) and 7-day sleep diaries (which were completed by participants at home and brought in for review by research staff) at baseline and all follow-up time points (i.e., post-treatment, 6-month follow-up and 12-month follow-up). The change in the following subjective sleep outcome measures obtained via questionnaires and sleep diaries was used to determine the efficacy of the CBT-I treatment: (1) sleep onset latency in minutes (the number of minutes it took for the participant to fall asleep after going to bed (diary sleep onset latency (SOL-D)); (2) diary wake after sleep onset (WASO-D) in minutes, which was the number of minutes the participant was awake during the night after falling asleep; (3) diary total wake time (TWT-D) at night in minutes, which was the total number of minutes awake between bedtime and rise time the next morning; and (4) sleep efficiency (which was the time asleep over the time in bed between bedtime and rise time the next morning) expressed as a percent (diary sleep efficiency, SE-D), all based on sleep diaries; (5) the PSQI (scores from 0 to 21; higher scores signify worse sleep; Buysse et al., 1989); and (6) the ISI (scores from 0 to 28; higher scores signify worse insomnia; Morin et al., 2011), collected in participant questionnaires. Sleep efficiency was also measured objectively using wrist actigraphy (SE-A), which was worn by participants for seven consecutive days and nights at each time point. The actigraph devices used were small watch-sized devices validated for use in longitudinal, naturalisticassessment of sleep (Actiwatch Spectrum, Philips Respironics; Morgenthaler et al., 2007). Blinded research staff scored actigraphy with validated software using defined “night” and “day” based on participant self-report of bedtimes and rising times on their sleep diaries for the days and nights they wore the wrist actigraph. These specific sleep variables were selected as outcomes in the current analysis since they were previously reported in the original randomized controlled trial to have significant improvement in the intervention group compared to the control group with the exception of SE-A, which was included as an objective measure of sleep (Alessi et al., 2016).

### Statistical Analysis

All analyses involved participants randomized to active treatment (CBT-I). Differences between physical activity groups at baseline were evaluated using two-sample *t*-tests for continuous variables and Fisher’s Exact tests for categorical variables. The seven sleep outcomes described above were analyzed with a two by four factorial mixed-effects model with a fixed intercept in which baseline physical activity was a two-level between-subjects factor (high vs. low physical activity), and time was a four-level repeated-measures factor (baseline, post-treatment, 6-months, 12-months). When used for repeated-measures designs, mixed-effects models allow for incomplete data for certain time points (i.e., to address dropouts and missing data; Littell et al., 2000). We used these mixed-effects models to calculate a mean and 95% confidence interval for each sleep outcome at each time point, as well as a difference in the average change from baseline to each follow-up time point, for each sleep outcome for the two physical activity groups.

Following the mixed-effects models, equivalence tests were performed to see if changes in sleep outcomes were the same in the two physical activity groups based on clinical significance thresholds established *a priori*. The equivalence tests were run to determine if the mean and 95% confidence interval for the difference between the physical activity groups in their changes from baseline for each sleep measure and to each time point (calculated by the mixed-effects model) were within pre-specified clinical significance thresholds. Clinical significance thresholds defined the difference in change needed between the two groups for them to be considered clinically different. If the confidence interval is completely within the threshold, then there is a high degree of certainty that the two groups are clinically the same. On the other hand, when the confidence interval overlaps the threshold, this does not prove differences between the groups. For this analysis, two thresholds of clinical significance were used (one larger and one smaller) to account for possible variation in clinical significance for CBT-I. For actigraphy and diary measures, a numeric value (i. e., minutes) was established for each threshold. For PSQI and ISI, thresholds were calculated using an effect size of 0.8 for the large threshold (large effect size) and 0.4 (half of the large threshold) for the small threshold (medium effect size; Cohen, 1992). Threshold values can be seen in Supplementary Table S1.

## Results

### Participant Characteristics

Among the 106 participants randomized to active treatment (i.e., CBT-I), 46 (43.4%) reported 3 days of physical activity per week or fewer (i.e., low physical activity group, *N* = 46), and 60 (56.6%) reported 4 days of physical activity per week or more (i.e., high physical activity group, *N* = 60). As shown in Table 1, the high physical activity group was significantly older (*P* = 0.049), scored higher on the SF-12 Physical Composite Subscale (i.e., had better health-related quality of life, *P* = 0.01), was more likely to have had sleep problems for 12 or more months (*P* = 0.02), was more likely to have less than a high school education (*P* = 0.04), was less likely to be divorced/separated (*P* = 0.001), and had fewer comorbidities (*P* = 0.02).

**Table 1:** Baseline characteristics of subjects.

Variable	Overall *N* = 106	Low activity *N* = 46	High activity *N* = 60	
	Mean (SD) or percent	Mean (SD) or percent	Mean (SD) or percent	Difference (*p*-value)
Age, in years	72.1 (7.9)	70.3 (7.9)	73.4 (7.7)	−3.0 (0.049)
Gender, male	96.2%	97.8%	95.0%	2.8% (0.63)
Race/ethnicity				
-Hispanic/Latino	6.6%	10.9%	3.3%	7.6% (0.24)
-Black/African American	5.7%	4.4%	6.7%	−2.3% (0.70)
-White	78.3%	80.4%	76.7%	3.7% (0.81)
Education				
-Less than high school	5.7%	0%	10.0%	10.0% (0.04)
-High school graduate	17.0%	19.6%	15.0%	4.6% (0.61)
-Some college	41.5%	43.5%	40.0%	3.5% (0.84)
-College graduate	17.0%	21.7%	13.3%	8.4% (0.30)
-Post baccalaureate	18.9%	15.2%	21.7%	−6.5% (0.46)
Marital status				
-Married	40.6%	30.4%	48.3%	−17.9% (0.08)
-Divorced/separated	32.1%	50.0%	18.3%	31.7% (0.001)
-Widowed	9.4%	6.5%	11.7%	−5.2% (0.51)
-Single/never married	10.4%	8.7%	11.7%	−3.0% (0.75)
Employment				
-Not working	76.4%	80.4%	73.3%	7.1% (0.49)
-Working part-time	17.9%	13.0%	21.7%	−8.7% (0.31)
-Working full-time	5.7%	6.5%	5.0%	1.5% (1.00)
Sleep diary measures				
-Sleep onset latency, in min	43.3 (47.9)	39.7 (37.7)	46.1 (54.6)	−6.4 (0.50)
-Wake after sleep onset, in min	55.8 (40.3)	49.6 (34.8)	60.6 (43.7)	−11.0 (0.16)
-Total wake time, in min	144.0 (89.5)	135.2 (83.9)	150.8 (93.7)	−15.6 (0.38)
-Sleep efficiency, in min	72.0 (14.8)	73.6 (13.3)	70.8 (15.8)	2.8 (0.33)
Sleep efficiency-actigraphy, in %	83.7 (6.1)	83.7 (5.5)	83.7 (6.5)	0.04 (0.97)
Pittsburgh Sleep Quality Index	9.4 (3.5)	10.0 (3.7)	9.1 (3.3)	0.9 (0.19)
Duration of sleep problems >12 months, % of subjects	90.6%	82.6%	96.7%	−14.1% (0.02)
Insomnia Severity Index	11.7 (5.3)	11.7 (4.9)	11.6 (5.6)	0.1 (0.96)
Apnea-hypopnea index	9.9 (5.4)	10.0 (5.0)	9.8 (5.6)	0.2 (0.86)
Comorbidity Index	7.0 (3.5)	7.9 (3.9)	6.3 (3.0)	1.6 (0.02)
Pain (GPM)	15.0 (11.6)	15.5 (10.9)	14.6 (12.2)	0.9 (0.70)
Depression (PHQ-9)	5.1 (4.3)	5.8 (5.0)	4.5 (3.7)	1.4 (0.11)
Quality of life (SF12-MCS)	52.9 (10.0)	51.3 (10.6)	54.1 (9.4)	−2.9 (0.14)
Quality of life (SF12-PCS)	44.2 (10.9)	41.2 (11.1)	46.5 (10.2)	−5.3 (0.01)
Epworth Sleepiness Scale	5.4 (4.0)	5.5 (4.2)	5.3 (4.0)	0.2 (0.79)
Flinders Fatigue Scale	9.8 (7.6)	10.6 (7.2)	9.2 (7.8)	1.4 (0.34)
DBAS-16	4.0 (2.1)	4.4 (2.2)	3.6 (1.9)	0.8 (0.05)

*GPM, Geriatric Pain Measure pain intensity subscale; PHQ-9, Patient Health Questionnaire-9; SF12-MCS and SF12-PCS, Medical Outcomes Study 12-item Short-form Survey v2 Mental and Physical Composite Subscales, respectively; DBAS-16, Dysfunctional Beliefs and Attitudes about Sleep*.

### Attrition

The analyses were intention to treat; no randomized participants were excluded from analyses. Of the 106 participants randomized to active treatment, 97 (91.5%), 92 (86.7%) and 89 (83.9%) completed post-treatment, 6-month and 12-month assessments, respectively. Attrition was not statistically different between the two physical activity groups at any assessment time point. At the 12-month follow-up, 82.6% of low activity participants and 85.0% of high activity participants completed assessment (*P* = 0.74).

### Outcomes

#### Mixed-Effects Models

**Figure 1:**
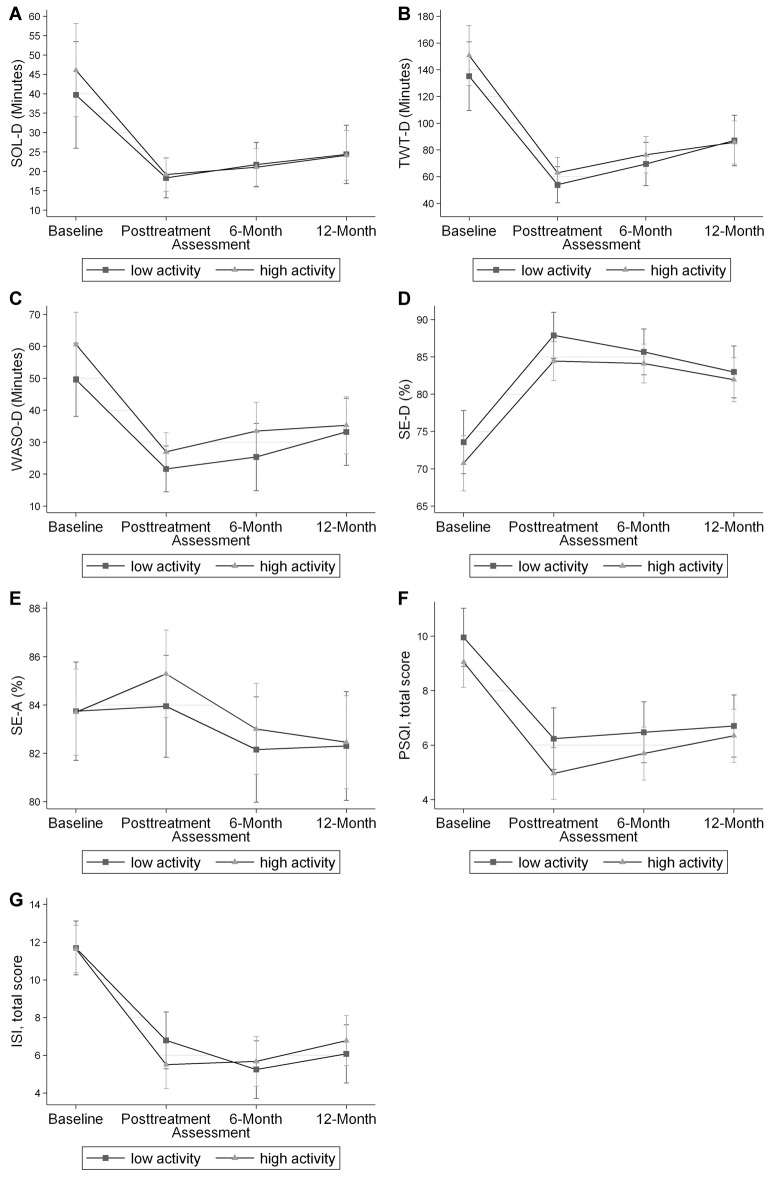
Mixed effects-models mean and 95% confidence interval for each sleep outcome at each time point. No differences were found for any of the sleep outcomes at each time point. Panel **(A)** shows the data for diary sleep onset latency (SOL-D) in minutes. Panel **(B)** shows the data for diary total wake time (TWT-D) in minutes. Panel **(C)** shows the data for diary wake after sleep onset (WASO-D) in minutes. Panel **(D)** shows the data for diary sleep efficiency (SE-D) in percent. Panel **(E)** shows the data for actigraphy sleep efficiency (SE-A) in percent. Panel **(F)** shows the data for the Pittsburgh Sleep Quality Index (PSQI), total score. Panel **(G)** shows the data for the Insomnia Severity Index (ISI) total score.

Table 2 shows the results of the mixed-effects models (with results shown graphically in Figure 1), which were used to calculate a mean and 95% confidence interval for each sleep outcome at each time point for the two physical activity groups. In these analyses, no differences were found between the physical activity groups for any sleep outcomes at any time point. Table 3 shows the results of mixed-effects models comparing the differences for each sleep outcome from the baseline to each time point, between the high and low physical activity groups. As indicated in the table, the *p*-values from the respective mixed-effects models did not demonstrate significant differences between the two physical activity groups.

**Table 2:** Sleep measures at baseline and follow-up by physical activity group.

Outcome	Mean (95% Confidence Interval)			
	Baseline assessment	Posttreatment assessment	6-Month assessment	12-Month assessment
Diary sleep onset latency (SOL-D), minutes				
Low PA	39.7 (26.0–53.4)	18.3 (13.1–23.5)	21.7 (16.0–27.5)	24.4 (16.8–31.9)
High PA	46.1 (34.1–58.1)	19.2 (14.8–23.5)	21.1 (16.2–25.9)	24.1 (17.7–30.6)
Diary wake after sleep onset (WASO-D), minutes				
Low PA	49.6 (38.1–61.1)	21.6 (14.5–28.8)	25.4 (14.8–35.9)	33.2 (22.8–43.7)
High PA	60.6 (50.6–70.7)	27.0 (21.0–33.0)	33.5 (24.6–42.5)	35.3 (26.3–44.2)
Diary total wake time at night (TWT-D), minutes				
Low PA	135.2 (109.5–160.8)	54.0 (40.5–67.6)	69.5 (53.4–85.7)	87.1 (68.2–106.0)
High PA	150.8 (128.3–173.2)	63.0 (51.6–74.4)	76.4 (62.7–90.2)	85.7 (69.6–101.7)
Diary sleep efficiency (SE-D), %				
Low PA	73.6 (69.3–77.8)	87.9 (84.8–91.0)	85.7 (82.6–88.8)	83.0 (79.5–86.5)
High PA	70.8 (67.0–74.5)	84.4 (81.8–87.0)	84.1 (81.5–86.7)	81.9 (79.0–84.9)
Actigraphy sleep efficiency (SE-A), %				
Low PA	83.7 (81.7–85.8)	84.0 (81.8–86.1)	82.2 (80.0–84.3)	82.3 (80.1–84.6)
High PA	83.7 (81.9–85.5)	85.3 (83.5–87.1)	83.0 (81.1–84.9)	82.5 (80.5–84.4)
Pittsburgh Sleep Quality Index (PSQI), total score				
Low PA	10.0 (8.9–11.0)	6.2 (5.1–7.4)	6.5 (5.4–7.6)	6.7 (5.6–7.8)
High PA	9.1 (8.1–10.0)	5.0 (4.0–5.9)	5.7 (4.7–6.7)	6.3 (5.4–7.3)
Insomnia Severity Index (ISI), total score				
Low PA	11.7 (10.3–13.1)	6.8 (5.3–8.3)	5.2 (3.7–6.8)	6.1 (4.5–7.6)
High PA	11.6 (10.4–12.9)	5.5 (4.2–6.8)	5.7 (4.4–7.0)	6.8 (5.5–8.1)

*(N = 46 Participants With Low PA, N = 60 Participants With High PA)*.

**Table 3:** Results of mixed-effects models comparing difference in change in sleep outcomes from baseline to each follow-up between high (*N* = 60) and low (*N* = 46) physical activity groups.

Outcome	Difference between groups in change from baseline (95% Confidence Interval)	*p*-value
Diary sleep onset latency (SOL-D), minutes		
Posttreatment assessment	−5.5 (−24.0–12.9)	0.56
6 months	−7.1 (−23.6–9.5)	0.40
12 months	−6.6 (−23.3–10.0)	0.43
Diary wake after sleep onset (WASO-D), minutes		
Posttreatment assessment	−5.7 (−19.2–7.8)	0.41
6 months	−2.9 (−18.4–12.6)	0.71
12 months	−9.0 (−26.5–8.5)	0.31
Diary total wake time at night (TWT-D), minutes		
Posttreatment assessment	−6.7 (−40.6–27.2)	0.70
6 months	−8.7 (−39.2–21.8)	0.58
12 months	−17.1 (−50.9–16.8)	0.32
Diary sleep efficiency (SE-D), %		
Posttreatment assessment	−0.6 (−6.6–5.3)	0.83
6 months	1.3 (−4.3–6.8)	0.66
12 months	1.8 (−4.5–8.0)	0.58
Actigraphy sleep efficiency (SE-A), %		
Posttreatment assessment	1.4 (−1.0–3.7)	0.25
6 months	0.9 (−1.6–3.4)	0.48
12 months	0.2 (−2.4–2.8)	0.88
Pittsburgh sleep quality index (PSQI), total score		
Posttreatment assessment	−0.4 (−1.8–1.0)	0.61
6 months	0.1 (−1.3–1.6)	0.86
12 months	0.5 (−0.9–2.0)	0.46
Insomnia severity index (ISI), total score		
Posttreatment assessment	−1.2 (−3.4–0.9)	0.26
6 months	0.5 (−1.7–2.7)	0.66
12 months	0.8 (−1.4–3.0)	0.49

#### Equivalence Tests

**Figure 2:**
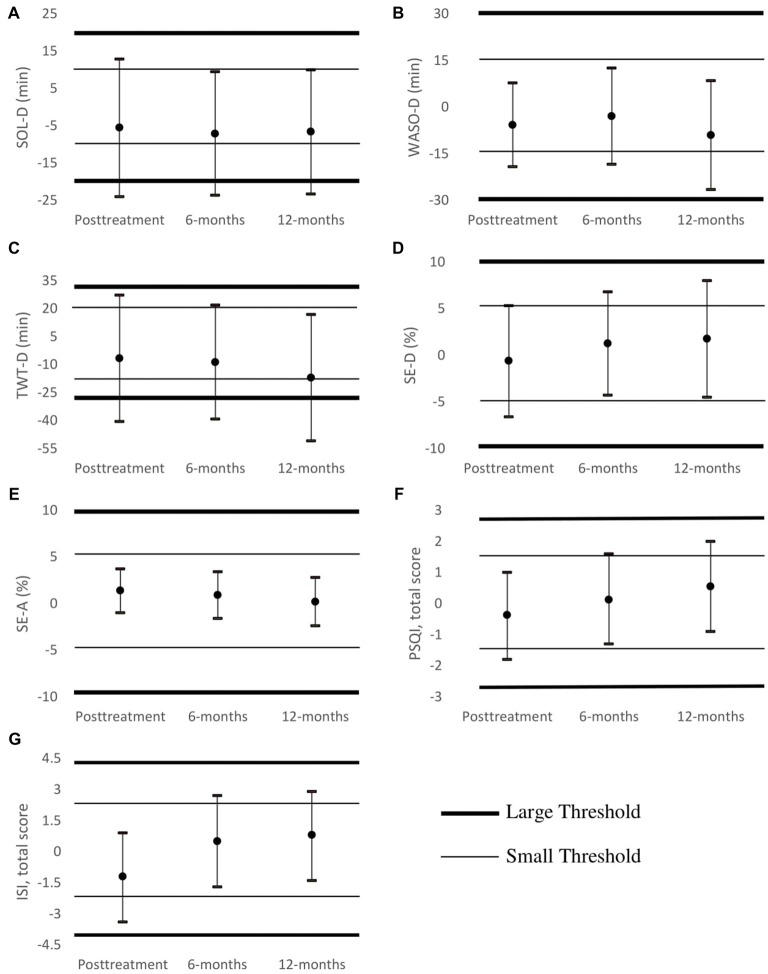
Equivalence tests using both thresholds of clinical significance (bold line for large threshold, thin line for small threshold) at post-treatment, 6- and 12-month follow-ups. Groups are equivalent if confidence interval brackets are completely within clinical thresholds (depicted as horizontal lines). Panel **(A)** shows that SOL-D in minutes was not significantly equal for the two groups by either threshold. Panel **(B)** shows that WASO-D in minutes was equal for the two groups using the large threshold but not the small threshold. Panel **(C)** shows that TWT-D in minutes was not equal by either threshold. Panel **(D)** shows SE-D in percent was equal using the large threshold but not the small threshold. Panel **(E)** shows SE-A in percent was equal for the PA groups by both thresholds. Panel **(F)** shows PSQI total score was equal using the large threshold but not the small threshold. Panel **(G)** shows ISI total score was equal using the large threshold but not the small threshold.

Equivalence tests were performed to see if changes between the two physical activity groups in sleep outcomes were the same based on clinical significance thresholds established *a priori*. Figure 2 shows means and 95% confidence intervals in relation to clinical significance thresholds. The difference in change in WASO-D, SE-D, SE-A, PSQI and ISI from baseline to each follow-up was within the large clinical significance thresholds. However, when held to small clinical significance thresholds, only SE-A fell within the pre-specified thresholds. Treatment effects in the two physical activity groups were overall within the large but not the small clinical thresholds.

## Discussion

We examined the relationship between baseline physical activity and sleep outcomes with CBT-I among older adults participating in a randomized controlled trial, who met diagnostic criteria for insomnia, and were randomized to receive an intervention involving CBT-I. We hypothesized that low baseline physical activity would adversely impact the efficacy of CBT-I, and participants who had higher levels of baseline physical activity would have better sleep outcomes compared to those who had lower levels of baseline physical activity. Surprisingly, we found that for the sleep outcomes we measured, physical activity level at baseline did not have a significant effect on the efficacy of CBT-I in older veterans at any follow-up time point, for up to 12 months follow-up. Contrary to our hypotheses, less physical activity at baseline did not predict a significantly smaller treatment effect from CBT-I. In addition, with several sleep outcomes, the differences between physical activity groups were within large clinical significance thresholds (suggesting equivalence of effect between high and low physical activity groups).

These findings suggest that CBT-I is similarly effective in older adults with lower baseline physical activity as among those with higher levels of baseline physical activity. In both the high and low physical activity groups, the expected trend of improved sleep outcomes in all sleep measures was observed, indicating improvement in sleep outcomes with CBT-I. For example, SOL-D decreased from baseline to all subsequent time points in both high and low physical activity groups, with no significant variation between the two groups. This information suggests that physicians should recommend CBT-I to older adults with insomnia disorder, even if their activity levels are low.

Mixed-effects models found no statistical difference between the two physical activity groups for any of the sleep outcomes. However, this did not prove that the two physical activity groups were equivalent. Thus, we performed equivalence tests for the differences in changes in sleep outcomes from baseline to each time point for each sleep outcome to see if the two physical activity groups were indeed clinically equivalent. Equivalence tests are highly dependent on the threshold defining clinical significance, and we understand that different clinical significance thresholds could yield varying results. Thus, two thresholds were established. When a large threshold was used (see Figure 2), meaning larger values were required to consider the two groups different, we found that the two physical activity groups were the same in five of the seven sleep outcomes. When a small threshold was used (see Figure 2), only one of the seven variables was found to be equivalent. These results do not clearly establish whether CBT-I is equally effective for the two physical activity groups because the degree to which baseline physical activity may affect CBT-I outcomes in part depends on the definition of what the patient or provider considers clinically significant. Regardless, none of the tests impugn the effectiveness of CBT-I for either physical activity group as the patterns in the outcome means as seen in Figure 1 are highly parallel. Furthermore, there was a large amount of variation in the width of the confidence intervals for many outcomes, making it more difficult to detect and assert nonequivalence. In fact, some of the confidence intervals, such as those for SOL-D and TWT-D, were wider than the threshold itself.

The other findings from these analyses were as we expected. For example, the low physical activity group had more comorbidities than the high physical activity group since additional health problems often contribute to low physical activity (Durstine et al., 2013), and moderate physical activity helps prevent certain chronic diseases (Kruk, 2007). It was also not surprising that the low physical activity group had on average a lower SF12-PCS score, suggesting that those with less physical activity had lower physical ability and quality of life. There was no difference in completion of the study by activity group, meaning that those with low baseline physical activity were not more likely to drop out of the treatment program, so differences in loss to follow-up between groups likely do not explain these results.

We believe these findings are promising and important for older patients who have insomnia, and low physical activity levels. The efficacy of CBT-I was present in both low and high physical activity groups. In addition, regardless of physical activity level at baseline, similar trends were seen in all graphs in Figure 1, suggesting those with lower baseline physical activity levels still benefitted from CBT-I.

Some limitations of our study should be considered. For example, this was secondary analysis, so the original study was not designed to test the effect of physical activity at baseline on CBT-I outcomes. Baseline physical activity level was determined by a self-reported question only, and more detailed information on the time of day, duration, or intensity of their physical activity was not collected. Future research should consider use of more detailed self-reported data on baseline physical activity, in addition to objective measures (e.g., portable devices designed to measure physical activity). Also, the limited sample size of the two physical activity groups could have impacted our ability to identify statistically significant differences. There were some differences in subjects included in this study, compared to the general US population of older adults. For example, this subject population had a greater percentage of active older adults compared to the national average (57% vs. 30%; Centers for Disease Control and Prevention, 2017), which may be in part due to the requirement for participants to attend treatment sessions and complete questionnaires at the facility as part of their participation in research. In addition, participants’ status as veterans may not generalize to all older people. Furthermore, the sample was mostly non-Hispanic white males, so these results may not be applicable to all populations of older adults.

Future research is needed to more fully understand the relationship between physical activity and outcomes of CBT-I. The lack of an association between baseline physical activity and efficacy of CBT-I may further be due to the adaptability of CBT-I recommendations to individual patient needs and abilities. Future work should directly address the relationship between changes in physical activity (rather than only at baseline) over the course of CBT-I treatment. Also, testing the effect of a more prominent physical activity component to CBT-I on the improvement of sleep outcomes in those with low baseline physical activity levels could prove to be beneficial.

In conclusion, we found no clinically important differences in sleep outcomes from CBT-I among those with low vs. high levels of baseline physical activity. These findings suggest that CBT-I should be considered for the management of insomnia among older adults, regardless of their level of physical activity.

## Author Contributions

TY, CA, JM, CF, LF, JD, YS and KJ contributed to the conception and design of the study. SJ organized the database. TY, MM and SJ performed the statistical analysis. TY wrote the first draft of the manuscript. CA wrote sections of the manuscript. All authors contributed to manuscript revision, read and approved the submitted version.

## Conflict of Interest Statement

The authors declare that the research was conducted in the absence of any commercial or financial relationships that could be construed as a potential conflict of interest.
