# Supplementary material for: Sleep Outcomes With Cognitive Behavioral Therapy for Insomnia Are Similar Between Older Adults With Low vs. High Self-Reported Physical Activity
Source: Front Aging Neurosci. 2018 Sep 13;10:274. doi: 10.3389/fnagi.2018.00274 (PMC6146107; doi:10.3389/fnagi.2018.00274)
Supplement: Supplementary file 1 [file Table_1.DOCX]

Supplementary Material

Baseline Physical Activity and the Efficacy of Cognitive Behavioral Therapy for Insomnia in Older Adults: Is Low Physical Activity Associated with Worse Sleep Outcomes?

**Timothy Yeung^a^; Jennifer L. Martin, PhD^b, c^; Constance H. Fung, MD, MSHS^b, c^; Lavinia Fiorentino, PhD^d^; Joseph M. Dzierzewski, PhD^e^; Juan C. Rodriguez Tapia, MD^c, f^; Yeonsu Song, PhD, RN^b, c^; Karen Josephson, MPH^b^; Stella Jouldjian, MSW, MPH^b^; Michael N. Mitchell, PhD^b^; and Cathy Alessi, MD^b, c^***

^a^ Washington & Jefferson College, Washington, PA USA

^b^ Geriatric Research, Education and Clinical Center; VA Greater Los Angeles Healthcare System; North Hills, CA, USA

^c^Department of Medicine, David Geffen School of Medicine, University of California at Los Angeles, Los Angeles, CA, USA

^d^Department of Psychiatry, University of California at San Diego, San Diego, CA, USA

^e^Department of Psychology, Virginia Commonwealth University, Richmond, VA, USA

^f^Department of Medicine, Pontificia Universidad Catolica de Chile, Santiago, Región Metropolitana, Santiago, Chile

*** Correspondence:**Cathy Alessi, MD

cathy.alessi@va.gov

# Supplementary Table

Supplementary Table 1. Clinical Threshold Values Established A Priori for Equivalence Tests.

| **Sleep Outcome** | **Clinical Threshold Values** | |
| --- | --- | --- |
|  | **Large Threshold** | **Small Threshold** |
| Diary sleep onset latency (SOL-D) in minutes | 20 | 10 |
| Diary wake after sleep onset (WASO-D) in minutes | 30 | 15 |
| Diary total wake time (TWT-D) in minutes | 30 | 20 |
| Diary and actigraphy sleep efficiency (SE-D, SE-A) in percent | 10 | 5 |
| Pittsburgh Sleep Quality Index (PSQI), total score | 2.8 | 1.4 |
| Insomnia Severity Index (ISI), total score | 4.2 | 2.1 |
